# Evidence-based policymaking when evidence is incomplete: The case of HIV programme integration

**DOI:** 10.1371/journal.pmed.1003835

**Published:** 2021-11-09

**Authors:** Jan A. C. Hontelez, Caroline A. Bulstra, Anna Yakusik, Erik Lamontagne, Till W. Bärnighausen, Rifat Atun

**Affiliations:** 1 Heidelberg Institute of Global Health (HIGH), Heidelberg University Medical Center, Heidelberg, Germany; 2 Department of Public Health, Erasmus MC, Erasmus University Medical Center Rotterdam, Rotterdam, the Netherlands; 3 The Joint United Nations Programme on HIV/AIDS (UNAIDS), Geneva, Switzerland; 4 Aix-Marseille School of Economics, CNRS, EHESS, Centrale Marseille, Aix-Marseille University, Les Milles, France; 5 Africa Health Research Institute (AHRI), Mtubatuba, South Africa; 6 Harvard Center for Population and Development Studies, Harvard University, Cambridge, Massachusetts, United States of America; 7 Department of Global Health and Population, Harvard T.H. Chan School of Public Health, Boston, Massachusetts, United States of America

## Abstract

Jan Hontelez and co-authors discuss the use of different types of evidence to inform HIV program integration.

**Summary points**

- Sustainable Development Goal 3 aims to “ensure healthy lives and promote well-being for all at all ages” and has set a target of achieving global universal health coverage, representing a major policy shift away from mostly disease-specific “vertical programmes”.
- While health service integration can be a promising strategy to improve healthcare coverage, health outcomes, and efficiency, the exact impact of integration in different settings is hard to predict, and policy makers need to choose from a large variety of integration strategies and opportunities with varying levels of scientific evidence.
- Using the case of health service integration for HIV in low- and middle-income countries, we outline implementation strategies for integration opportunities with lacking or scarce high-level causal evidence, based on existing frameworks and methodologies from within and beyond healthcare and implementation science.
- Proper use of scientific evidence in other contexts requires adequate and systematic assessments of the transportability of an intervention. Several methods exist that allow for judging transferability and comprehensively identifying key context-specific indicators across studies that can affect the reported impact of interventions.
- When (transferable) evidence is absent, we propose that by drawing on well-established design and implementation methodologies—underpinned by ongoing learning and iterative improvement of local service delivery strategies—countries could substantially improve decision-making even in the absence of scientific evidence.
- Reaching the goal of making the HIV response an integral part of a larger, universal, people-centred health system that meets the needs and requirements of citizens can be facilitated by applying lessons learned from implementation science and novel design methodologies.

## Introduction

The Sustainable Development Goal (SDG) 3 aims to “ensure healthy lives and promote well-being for all at all ages” [1,2] and has a target of achieving universal health coverage (UHC) by 2030 (SDG 3.8), where all people should have access to affordable and high-quality health services [3]. SDG 1, which aims to “end poverty in all forms everywhere,” has a target (SDG 1.3) to “implement nationally appropriate social protection systems and measures for all,” which requires availability of fiscal space for health and social programmes [2]. The SDGs represent a major policy shift away from mostly disease-specific and often official development assistance–supported “vertical programmes” in health, towards people-centred comprehensive approaches to healthcare [4]—where health systems are oriented towards promoting health equity by “leaving no one behind” while meeting all essential healthcare needs under the UHC umbrella [5].

Policy makers now face the daunting challenge of proposing, adapting, and implementing healthcare interventions tailored to specific contexts and population needs, often with limited high-quality scientific evidence on the impact of candidate interventions. Although evidence-based medicine remains the gold standard for decision-making in healthcare [6], randomised controlled trials of health system interventions are extremely time-consuming and often have low external validity because their results are context-specific [7]. As part of the PLOS collection on Joint United Nations Programme on HIV/AIDS (UNAIDS) HIV targets [8], we use the case of health service integration for HIV in low- and middle-income countries (LMICs) to outline existing frameworks and provide guidance on how to best utilise the existing evidence in policymaking and, in particular, how to design implementation strategies for integration opportunities where high-level causal evidence is scarce or lacking.

## The current global evidence base on HIV service integration

A recent comprehensive systematic review that was undertaken as part of the UNAIDS 2021–2025 HIV/AIDS target estimations and resource needs exercise, analysed and synthesised the existing evidence on the impact of integration of HIV services with other health services [9]. The findings of this study indicate that integrated services were mostly associated with better outcomes across a wide range of cascades-of-care and health indicators and provide a comprehensive new evidence base for policy. However, the wide variation of implementation design and contexts of implementations across the studies severely restricts the unadapted transfer of the findings to other settings and reveal gaps in the evidence base for promising integration opportunities and in specific geographical settings.

## Judging transferability of the existing evidence

Suitability of evidence for decision-making depends on both the quality of the evidence and the usefulness for a new context: Results from experiments in one context may or may not be useful for policy decisions in another context. As a context is defined by different determinants, which can be measured and accounted for to some extend (for instance, geographical location, healthcare infrastructure, and economic, political, and social environments [10]), in the majority of situations, generalizability to the current location and situation needs to be carefully considered, and study findings need to be adapted.

Different methods could be applied for judging overall transferability of the available evidence (**Table 1**). First of all, transferability should be judged by local policymakers, experts, and other stakeholders. How does the context in which the evidence for a certain intervention is generated differ from the context in which a policy decision need to be made, for instance, in terms of epidemiology and disease burden, target population, financial and human resources, health services in place, and targets for control? Mehrotra and colleagues (2019) propose a “transportability framework” to understand HIV programme effects in different contexts to enable judgement of external validity of interventions and translate this into policy action and testable insight [11]. Second, qualitative or mixed-method studies could be conducted to explore how tested interventions need to be adapted to be successful in the new setting [12,13]. Such a process involves a sequence of steps, generally consisting of (i) assessing the community and target population; (ii) evaluating the evidence base with local policymakers, experts, and other stakeholders; (iii) adapting the intervention for suitability in the local context; (iv) implementation; and (v) monitoring and evaluation [12]. Third, recent statistical methods—developed under the heading of “transportability studies”—such as robust targeted maximum likelihood estimators [14,15] and inverse odds of sampling weights [16] allow for predicting the impact of an intervention in a new setting.

**Table 1:** Conceptual framework for evidence-based decision-making on HIV service integration strategies by level of evidence.

Level of evidence	Evidence base, study types	Action
**Context-specific evidence on integration interventions**	• Local experimental study • Local implementation study	• Expert judgement • Epidemiological situation • Target population • Financial and human resources • Health systems in place
**Evidence on integration intervention from other contexts**	• Experimental studies from comparable context • Observational comparison studies from comparable contexts • Experimental or observational studies from distinctly different contexts and settings	• Assess likelihood that a proposed policy will be effective in achieving a predefined target and what type of evidence would be relevant for the evaluation [22,23] • Mechanism mapping [17] • Use “transportability framework” to judge transferability of the existing evidence; suitability for the context of interest [11] • Test transportability • Robust targeted maximum likelihood estimators [14,15] • Intervention adaptation [21] • For instance, service setting, target population, mode of delivery, cultural sensitivity
**No scientific evidence on impact of integration intervention available**	• Interventions implemented but no evidence of impact (yet) • Interventions not yet implemented and no experimental or observational studies from comparable contexts	• Hypothesise merit of intervention for local population • Deliberative ideation of promising integration opportunities based on local targets, populations, knowledge of barriers and enablers • Perform expert evidence survey to collect information about unpublished observations and case series for the context of interest [28] • Agile design and implementation • Systems-thinking processes [29] • Prototyping • Agile software development [32] • A/B testing [33,34] • Step-wedged randomised trials [35] • Iterative development cycles [29]

In attempting to understand, identify, and adapt interventions with problems of external validity simultaneously, Williams suggests “mechanism mapping” [17]—an approach in which the policy’s theory of change is juxtaposed with the underlying contextual assumptions needed for each step of this mechanism to operate and the actual characteristics of the policymaker’s context. By identifying specific aspects of the policy that are likely to be affected by the difference in context, the approach also directly informs intervention adaptation processes. However, such efforts require studies of health service delivery to report on important underlying factors in detail, something that is not always done explicitly in the published literature. Standardised reporting guidelines like the STROBE guidelines for observational studies or the STARI guidelines for implementation studies do call for describing the setting and generalisability of the findings [18,19], but adaptation and reinterpretation of the findings to suit local contexts requires a more comprehensive understanding of the contextual setting where the original study was performed [20,21]. For instance, Schloemer and colleagues found that 44 different criteria influence the transportability of health interventions, divided over 4 overarching themes: (i) population in which the intervention was studied; (ii) intervention characteristics; (iii) environmental characteristics; and (iv) the transferability process [13]. Maximising transportability of context-specific interventions thus requires a comprehensive, systematic approach to reporting key context-specific indicators across studies.

## Decision-making in the absence of scientific evidence

Transporting and adapting context-specific findings certainly enhances the usefulness of context-specific effect studies. Many promising integration opportunities, however, have limited to no quantitative scientific evidence on impact. This is especially true for key populations in the HIV response, such as transgender people, migrants, men who have sex with men, and sex workers; for particular geographic regions, such as Latin America, Southeast Asia, and the Russian Federation; and for specific disease-based opportunities for integration, such as cervical cancer, mental health, or schistosomiasis. By drawing on well-established design and implementation methodologies—underpinned by ongoing learning and iterative improvement of local service delivery strategies—countries could substantially improve decision-making even in the absence of scientific evidence (**Table 1**).

The scope of claims that require evidence should be judiciously made. Cartwright and Stegenga (2013) propose to approach the issue from the policy makers’ perspective and work backwards; assessing what the likelihood is that a proposed policy will be effective in achieving a predefined target and what type of evidence would be relevant for the evaluation [22,23].

There are many examples in which integration opportunities are straightforward, while the risks are likely negligible, allowing for implementation even without a solid evidence base. For instance, the integration of health services is often a sensible choice when the services are targeted at the same end users, like integrating HIV services and gender-affirming therapies for transgender men and women [24] or integrating HIV services and cervical cancer screen-and-treat strategies for HIV-infected women [25]. Also, in cases of HIV service integration with social programmes, we know many examples of positive impacts, and we can question whether we truly need to await the lengthy process of formally testing impact before we can implement these elsewhere. For instance, we know that adolescents can benefit from integration of youth-friendly health services with social programmes that include sex education and mental well-being awareness campaigns, like being done by the “Youth Hub” in Malawi [26] and the Zvandiri project in Zimbabwe [27].

When scientific evidence is lacking, but the intervention of interest is already implemented in a given or a comparable context, expert evidence surveys can be administered to local experts to gather information about their unpublished observations and case series as a basis for developing recommendations that are as evidence-based as possible at a particular point in time [28].

When scientific evidence is lacking and comparable interventions have yet to be implemented, implementation and testing of understudied integration opportunities can be aided by lessons learned in other sectors, such as business or software design. Applying systems-thinking processes to integrating the HIV response, for instance, by framing problem definitions and applying context analyses, ideation, creative thinking, prototyping, testing, and evaluation, could ensure that the right solutions to context-specific problems are generated and iteratively refined [29]. Similarly, widely applied agile processes in service sectors, such as software development [30], which emphasise the need to collaborate with customers and respond to changes in demand in an agile way, seem to be highly relevant and applicable when redesigning health systems in the absence of clear scientific knowledge that reflects the rapidly changing contexts. Applying incremental, iterative development cycles, in which a system is gradually or radically improved as new evidence is emerging, could reduce the task of redesigning an entire health system to more manageable and feasible components that can be optimised individually [31].

## How to arrive at an integrated HIV response

For people-centred approaches to health systems integration to be successful, deliberative inclusive processes [36]—in which stakeholders, from international donors to local policy makers, service implementers, civil society organisations, and end users are involved as equal partners—should be at the core of decision-making. For instance, human-centred design studies, in which the needs and requirements of the users are systematically analysed, could help ensure that local needs and preferences are considered throughout the design and implementation process [37,38]. Co-creation, or co-design, is a deliberative process defined as the collaborative generation of knowledge by academics working alongside stakeholders from other sectors [38]. It builds on the foundation that key information and knowledge on underlying processes and determinants of success and failure are often well known by implementers and consumers.

While redesigning the HIV response to meet the changing contextual needs, we should also ensure that we accelerate the sharing of knowledge within the public domain. Approaches, such as “A/B testing” [33,34], i.e., experimental designs in which several variants of a new service are randomly offered to people and service uptake and effects are measured in real time, could rapidly result in knowledge on preferences, barriers, and enablers of specific health service design choices. Furthermore, health system innovations could contain built-in trial elements to ensure rapid impact assessment and knowledge generation alongside real-world implementation, for instance, through stepped-wedge randomised trials [35,39,40].

## Discussion

Health policy makers and funders are tasked with the daunting challenge of redesigning the HIV response to meet the current complex epidemiological, financial, and political challenges. By applying lessons learned and methods from a range of scientific fields—such as innovation models, systems dynamics models, implementation science, design research, and learning systems—policy makers, funders, and practitioners could help accelerate and maximise the transfer of limited, and often context-specific, knowledge on best practices for developing integrated HIV services. Furthermore, by expanding the evidence with results from widely applied methods that are not randomised controlled trials, policy makers could help ensure that the HIV response is an integral part of a larger, universal, people-centred health system that meets the needs and requirements of citizens. Such a shift in decision-making design could prove to be key in shaping the HIV response within the UHC agenda in rapidly changing contexts.

## Abbreviations

LMIC: low- and middle-income country
SDG: Sustainable Development Goal
UHC: universal health coverage
UNAIDS: Joint United Nations Programme on HIV/AIDS
